# AAV8-mediated CasRx delivery and expression in the cochlea and cochlear nucleus of mice

**DOI:** 10.1016/j.bbrep.2026.102829

**Published:** 2026-11-02

**Authors:** Shuang Zhou, Tao Huang, Wei Yao, Shan Huang, Junjie Jiang, Hua Liao, Zhongfang Xia

**Affiliations:** a Wuhan Children's Hospital (Wuhan Maternal and Children Healthcare Hospital), Tongji Medical College, Huazhong University of Science and Technology, PR China; b Renmin Hospital of Wuhan University, Wuhan, 430060, PR China

**Keywords:** AAV8, CasRx, RfxCas13d, Cochlea, Cochlear nucleus, Auditory safety, RNA-Targeting platform

## Abstract

CRISPR/Cas9‐based genome editing has made substantial progress in recent years and has enabled the treatment of a variety of genetic diseases, including hearing loss in congenitally deaf mice. However, successful gene editing for hereditary deafness may still induce off‐target mutations in genomic DNA, which limits its clinical translation. CasRx, also known as RfxCas13d, is a compact RNA-targeting CRISPR effector that can be packaged into AAV vectors for in vivo delivery. CasRx, a recently identified member of the type VI CRISPR system, modulates target RNA expression without altering DNA sequences, thereby avoiding permanent off‐target mutations and offering broad therapeutic potential. Here, we used adeno-associated virus serotype 8 (AAV8) to deliver CasRx into the cochlea and cochlear nucleus (CN) of C57BL/6J mice and evaluated its expression pattern and short-term safety. Two weeks after viral injection, the number and morphology of neurons and other cell types in the CN remained normal. Importantly, auditory brainstem response (ABR) thresholds were unchanged after viral delivery, indicating that AAV8-mediated CasRx expression did not detectably impair auditory function during the short observation period. In conclusion, this study demonstrates the feasibility and short-term safety of AAV8-mediated CasRx delivery and expression in the cochlea and cochlear nucleus, providing a delivery platform for future guide RNA-directed transcript-modulation studies in auditory disorders.

## Highlights

• CasRx enables RNA editing without introducing genomic DNA mutations.
• AAV8 mediated stable CasRx expression in mouse cochlea and cochlear nucleus.
• No adverse effects observed on neuronal morphology or ABR thresholds.
• Provides a safe RNA editing strategy for tinnitus and hearing loss therapy.

## 1 Introduction

According to the World Health Organization, more than 1.5 billion people worldwide live with some degree of hearing loss, and approximately 430 million require rehabilitation services, including 34 million children [1]. Sensorineural hearing loss (SNHL) is the most common type and arises from pathology in the inner ear, sensory epithelium, or auditory nerve [2]. To date, more than 100 genetic loci have been linked to hereditary hearing loss (http://hereditaryhearingloss.org/), and advances in molecular genetics have greatly improved understanding of the pathophysiology of deafness and the diagnosis of causative mutations. However, no effective pharmacological therapy is currently available for hereditary non‐syndromic SNHL. Although most patients with SNHL can obtain partial auditory function with hearing aids or auditory implants, limitations such as poor speech discrimination still exist [3]. Thus, novel and more effective therapeutic strategies are urgently needed.

Gene therapy, which introduces functional genes to restore or compensate for defective genes complement mutated or deleted alleles, has developed rapidly over the past decades [4]. LUXTURNA™, an AAV‐based gene therapy for RPE65 mutation–associated retinal dystrophy, has been approved by the U.S. Food and Drug Administration (FDA), highlighting the promise of viral gene delivery for genetic diseases [5]. In the field of hearing research, several groups have explored viral gene delivery and genome-editing approaches for hereditary hearing loss, particularly following the development of CRISPR-based technologies [6], [7], [8], [9], [10], [11], [12], [13], [14], [15], [16], [17]. Gao et al. delivered a Cas9-gRNA ribonucleoprotein complex targeting the mutant Tmc1 allele in Beethoven mice at P0-2 and protected auditory function during subsequent hearing development [18], representing the first successful application of gene editing to treat hearing loss. Similarly, Xue et al. used an AAV-PHP.eB vector to deliver SaCas9-KKH and sgRNA complexes in a *Myo6* mutant mouse model and rescued the development of hearing impairment [19]. These studies collectively demonstrate the rapid development of gene-editing approaches for hearing protection and hearing preservation.

Recent advances in inner-ear gene therapy have expanded beyond early viral delivery studies and now include AAV-mediated gene replacement, clinical OTOF gene therapy, DNA/base-editing strategies, and RNA-targeting approaches. DNA-editing approaches, including CRISPR/Cas9-mediated allele disruption and DNA base editing, have shown therapeutic potential in several hereditary hearing-loss models [13], [20]. These studies highlight the promise of genome editing in the inner ear, while also emphasizing the need to consider permanence, genomic off-target effects, delivery constraints, and mutation-specific applicability.

Gene replacement is well suited for loss-of-function disorders when the therapeutic coding sequence can be efficiently packaged and expressed in the appropriate auditory cell type. Recent AAV-mediated OTOF gene-replacement studies and clinical trials have further demonstrated the therapeutic potential of inner-ear gene therapy [9], [21]. Compared with gene replacement or permanent DNA editing, RNA-targeting strategies offer a complementary approach by modulating transcripts without permanently altering genomic DNA. CasRx, also known as RfxCas13d, is a compact RNA-targeting CRISPR effector suitable for AAV-based delivery. Its ability to regulate RNA transcripts without altering genomic DNA makes it a useful candidate tool for transcript-level modulation, although functional application requires target-specific guide RNAs and direct validation of RNA-level effects [22]. Importantly, functional CasRx-mediated RNA knockdown requires a target-specific guide RNA, whereas RNA base-editing approaches generally require recruitment or fusion of an ADAR deaminase domain. Therefore, direct validation of on-target and off-target transcript effects is required for functional RNA-modulation studies. For instance, CasRx has been applied to inhibit pancreatic ductal adenocarcinoma progression and to manipulate neural cell fate in mice [22]. In the auditory system, CRISPR/CasRx has recently been explored in hearing-loss models. Knockdown of *Htra2* mRNA by AAV–CasRx–gRNA delivery into the cochlea prevented aminoglycoside‐induced hearing loss with minimal off‐target and systemic side effects [23]. Similarly, Zheng et al. used AAV-PHP.eB to deliver CasRx and sgRNA to the neonatal cochlea and successfully improved hearing and hair‐cell survival in *Tmc1*^Bth^ mice [24]. However, some forms of hereditary hearing loss are primarily caused by lesions confined to inner hair cells (IHCs), and the use of AAV8 for CasRx delivery and expression in adult IHCs has not been well characterized. In this study, we used AAV8, which has been reported to preferentially infect IHCs in adult mice, to deliver CasRx into the cochlea and evaluated its expression pattern and short-term structural and auditory safety. The present study does not test guide RNA-directed transcript knockdown or ADAR-based RNA editing; instead, it evaluates AAV8-mediated CasRx delivery and expression as a prerequisite for future guide RNA-directed RNA-modulation studies.

Tinnitus is defined as the perception of sound in the absence of an external acoustic stimulus and is often described with variable intensity, loudness, and pitch. It is a highly distressing condition that can lead to anxiety, irritability, depression, and even self‐harm or suicide, severely impacting quality of life [25]. With population aging, lifestyle changes, increased stress, and environmental noise exposure, the incidence of tinnitus has risen in recent years [25]. However, effective treatments remain lacking. Experimental and clinical studies have shown that tinnitus can originate from neurons in both the dorsal cochlear nucleus (DCN) and ventral cochlear nucleus (VCN), which display an increased spontaneous firing rate (SFR) [26]. Previous work demonstrated that fusiform cells in the DCN become hyperactive in tinnitus models, associated with reduced activity of KCNQ2/3 (Kv7.2/3) channels. Other studies revealed that changes in hyperpolarization‐activated cyclic nucleotide-gated (HCN) channel activity in these cells also correlate with tinnitus. Therefore, upregulation or downregulation of genes such as *Kcnq2/3* and *Hcn* in fusiform cells may provide new avenues for tinnitus research and therapy. Here, we injected AAV8-CMV-CasRx-Flag-GFP into the cochlear nucleus (CN) and evaluated its expression and short-term safety as a preliminary delivery assessment to support future guide RNA-directed transcript-modulation studies in CN circuits.

## 2 Materials and methods

### 2.1 Animal procedures

All animal experiments were conducted in qualified facilities and approved by the Animal Care and Use Committee of the People's Hospital of Wuhan University (approval No. 20220301A). Only male C57BL/6J mice were used in this initial feasibility and safety study to reduce variability in the small experimental cohort. Four‐ to five‐week‐old male C57BL/6J mice were purchased from Beijing Vital River Laboratory Animal Technology Co., Ltd. At least three mice were included in each group. All procedures followed the 3R principles of animal research (Reduction, Replacement, and Refinement). Wild-type male C57BL/6J mice were randomly assigned to the experimental groups according to the study design. After injection, 4–5 mice were housed per cage, and the animal room was maintained on a 12 h light/dark cycle with lights on from 08:00 to 20:00.

### 2.2 Production of virus

AAV vectors of different serotypes, including AAV8-GFP and AAV8-CasRx-Flag-GFP, were obtained from ViGene Biosciences (Shandong, China). Both vectors contained a GFP reporter, and transgene expression was driven by the CMV promoter [27], [28]. The titers of AAV8-GFP and AAV8-CasRx-Flag-GFP were 1.62 × 10^13 and 1.55 × 10^13 GCs/mL, respectively.

### 2.3 Microinjection into the inner ear of adult mice

Adult C57BL/6J mice were used for AAV injections via canalostomy. Mice were anesthetized with 5% chloral hydrate administered by intraperitoneal injection. The right postauricular region was shaved and disinfected with 10% povidone–iodine before surgery. A 10‐mm postauricular incision was made, and the right pinna and sternocleidomastoid muscle were bluntly dissected to expose the posterior semicircular canal (PSCC). The PSCC was perforated using a Bonn microprobe (Fine Science Tools, Foster City, CA) and connected to a micromanipulator (RWD) via a glass micropipette and a fine polyimide tube. The tip of the tube was inserted into the PSCC, and the opening was sealed with tissue adhesive (3 M Vetbond, St. Paul, MN) to allow AAV delivery. The total injection volume was 1 μL per ear at a rate of 100 nL/min. After injection, the tubing was cut and the puncture site was sealed with tissue adhesive. Mice were monitored until full recovery from anesthesia. Perioperative analgesia was provided using buprenorphine at 0.05 mg/kg subcutaneously 30 min before surgery and every 12 h for 48 h after surgery, together with meloxicam at 5 mg/kg subcutaneously immediately after surgery and once daily for 2 days, according to the approved animal protocol [29].

### 2.4 Microinjection into the cochlear nucleus of adult C57BL/6J mice

For cochlear nucleus injection, 4-week-old C57BL/6J mice were anesthetized and placed in a prone position. After shaving and disinfection of the scalp, a small posterior cranial opening was made, and the left cerebellar surface was exposed. Partial aspiration of the left cerebellum was carefully performed under a surgical microscope to directly visualize the cochlear nucleus region. The dorsal cochlear nucleus and adjacent ventral cochlear nucleus were identified based on their anatomical position at the lateral brainstem surface. A pulled glass micropipette with a final outer diameter of approximately 10 μm was mounted on a micromanipulator and advanced into the exposed CN region. AAV8-CMV-CasRx-Flag-GFP was injected unilaterally into the left CN at a volume of 2 μL per animal and a rate of 300 nL/min. The titers of AAV8-CMV-GFP and AAV8-CMV-CasRx-Flag-GFP were 1.62×10^13 GC/mL and 1.55×10^13 GC/mL, respectively. For cochlear injection, 1 μL virus was delivered per ear, corresponding to 1.62×10^10 GC/ear for AAV8-CMV-GFP and 1.55×10^10 GC/ear for AAV8-CMV-CasRx-Flag-GFP. For CN injection, 2 μL AAV8-CMV-CasRx-Flag-GFP was delivered per animal, corresponding to 3.10×10^10 GC/CN. After injection, the micropipette was kept in place briefly to minimize reflux and then slowly withdrawn. Successful targeting was verified post hoc by detecting Flag/GFP-positive cells in CN sections, including the DCN, PVCN, and AVCN.

### 2.5 Auditory testing

Two weeks after AAV injection, ABRs were recorded using the TDT System 3 (Tucker-Davis Technologies). Mice were anesthetized with 5% chloral hydrate by intraperitoneal injection at a dose of 400 mg/kg. During ABR testing, body temperature was maintained using a heating pad. Three subcutaneous needle electrodes were inserted: an active electrode dorsally behind the pinna, a reference electrode at the midline between the two ears, and a ground electrode dorsally at the rump near the tail.

### 2.6 Immunohistochemistry

Cochleae and CNs were harvested two weeks after AAV injection, cleaned, and fixed in 4% paraformaldehyde at 4°C overnight. Samples were decalcified in 120 mM EDTA (pH 7.4) for 2–3 h, blocked with 5% bovine serum albumin (BSA) for 1 h, and incubated overnight at room temperature with primary antibodies: anti‐MYO7A (1:300, Proteus Biosciences), anti‐Flag (1:200, Beyotime, AF519), and anti‐NeuN (1:200, Synaptic Systems, 135203). Tissues were washed three times with PBS and incubated with Alexa Fluor–conjugated secondary antibodies (1:500; Invitrogen), including donkey anti‐rabbit Alexa Fluor 555 and donkey anti‐mouse Alexa Fluor 488, for 1 h at room temperature. Confocal images were acquired using a Zeiss LSM 880 laser scanning confocal microscope. Tonotopic cochlear maps were generated using an ImageJ plugin [30].

### 2.7 Cell counting and quantification

Z‐stacks were acquired for each segment and converted to maximum intensity projections using ImageJ to quantify IHCs, outer hair cells (OHCs), NeuN + cells, and Flag + cells. We counted MYO7A + hair cells, MYO7A/GFP double‐positive cells, and Flag/NeuN double‐positive cells in cochlear whole mounts. Cells were counted in 20× fields of view at the apical, middle, and basal turns of the cochlea. Three animals per group were analyzed to determine transduction efficiency.

### 2.8 Statistical analysis

Data were analyzed using GraphPad Prism v8.0 (GraphPad Software, San Diego, CA). One‐way ANOVA with Bonferroni post hoc correction was used to compare ABR thresholds, hair‐cell survival, and viral transduction efficiencies. P values < 0.05 were considered statistically significant.

## 3 Results

### 3.1 Plasmid construction and expression

We synthesized the CasRx coding sequence and cloned it into a mammalian expression vector under the control of the CMV promoter. A Flag tag was fused to the C terminus of CasRx, and an EGFP reporter was linked via a P2A self‐cleaving peptide to enable independent expression (Fig. 1C). To assess CasRx expression, the plasmid was transfected into cultured HEK293T cells. Three days after transfection, robust EGFP fluorescence was observed by immunofluorescence microscopy, confirming efficient transfection (Fig. 1A). Western blot analysis of cell lysates detected strong expression of CasRx–Flag at the expected molecular weight (∼120 kDa) three days after plasmid delivery. The positive control expressing only Flag produced a smaller protein band, whereas no Flag signal was detected in the negative control. Together, these data demonstrate successful plasmid construction and efficient CasRx expression in vitro.

**Fig. 1:**
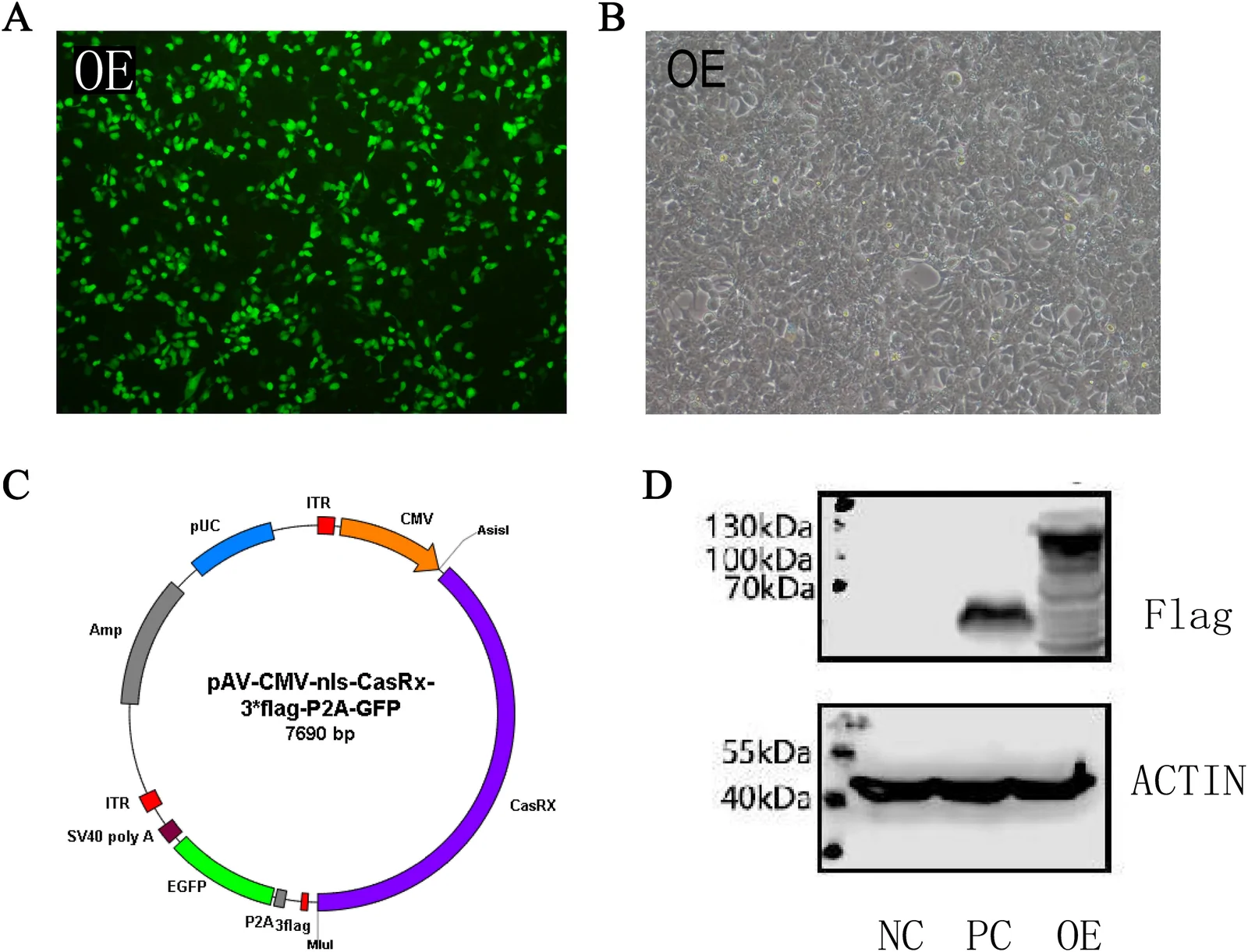
**CasRx Plasmid construction and transfection.** OE indicates HEK293T cells transfected with the CasRx-Flag-GFP overexpression plasmid. NC indicates non-transfected negative control cells, and PC indicates positive control cells expressing Flag. Representative GFP fluorescence and bright-field images are shown three days after transfection. Western blotting confirmed CasRx-Flag expression at approximately 120 kDa. (A) CasRx Plasmid transfected HEK293 cell lines. Three days after transfection, immunofluorescence showed infected cells were identified by native GFP. (B) Three days after transfection, Cell morphology remains normal. (C)Schematic of plasmid construction. (D) Western blot detected CasRx-Flag expression (120 kD). Representative images from 3 independent transfection experiments are shown.

### 3.2 Transduction of AAV8–GFP in the inner ear of adult mice

Based on previous work showing that AAV8 efficiently and specifically infects adult mouse IHCs [31], [32], [33], we selected AAV8 as the delivery vector. Because CMV is among the most efficient promoters for inner‐ear expression [27], it was used to drive transgene expression in vivo (Fig. 2A). After virus production, the titer of AAV8–CMV–GFP was determined to be 1.62 × 10^13 genome copies (GCs)/mL (Fig. 2B). We then injected 1 μL of AAV8–CMV–GFP into the inner ear of 4–8‐week‐old wild‐type mice via the posterior semicircular canal (PSCC) route, which is considered a safe and effective approach for inner‐ear gene delivery in adult mice [34]. Two weeks after injection, cochleae were harvested for immunostaining (Fig. 2C). The results showed that AAV8 exhibited high specificity for IHCs: GFP expression was detected exclusively in IHCs, whereas outer hair cells (OHCs) were GFP negative (Fig. 2C).

**Fig. 2:**
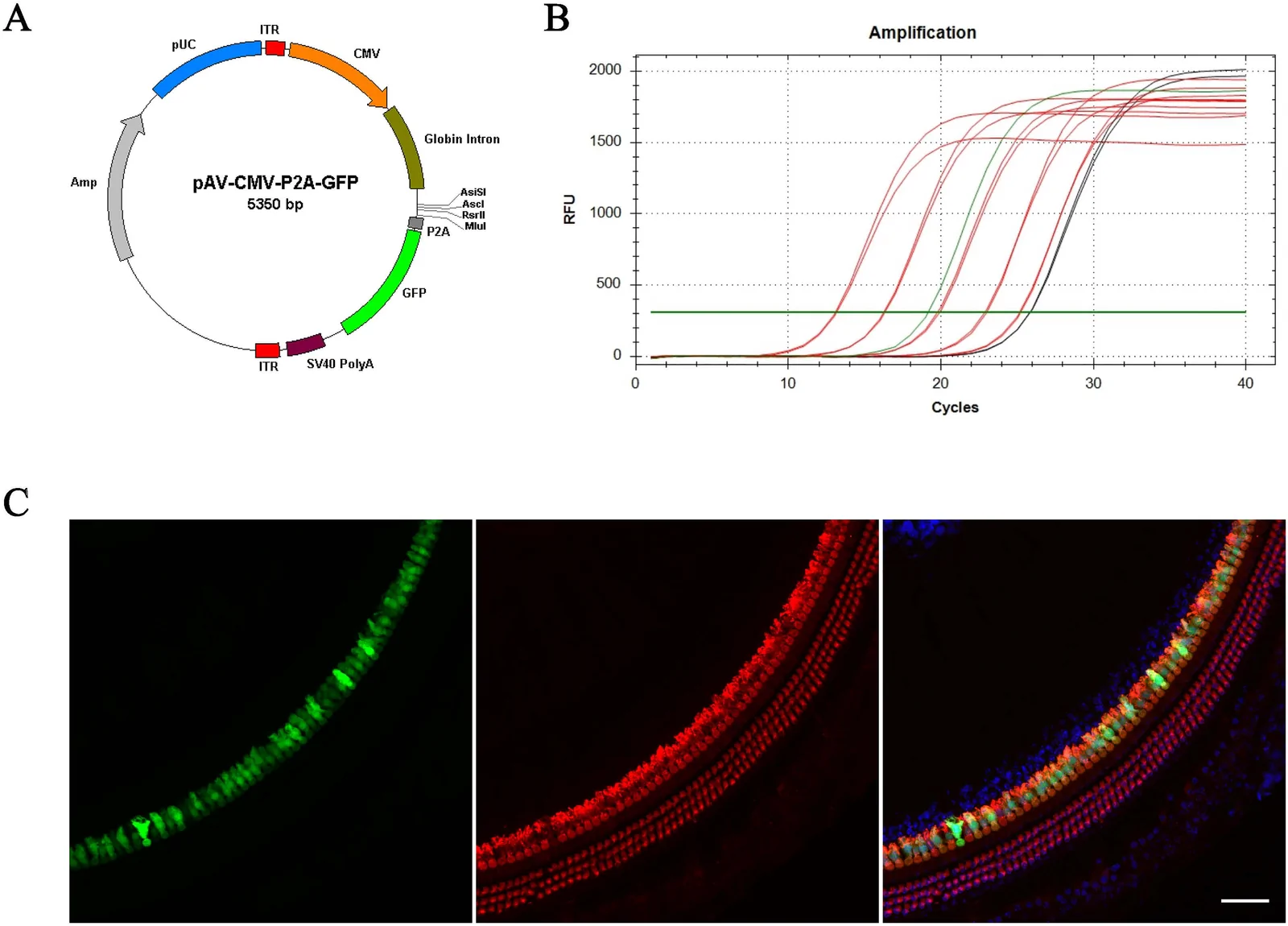
**AA8-CMV-GFP was produced and infected inner hair cells in mature cochleae.** (A) Schematic of the AAV8-CMV-GFP construct. (B) Viral genome titration assay used to determine the titer of AAV8-CMV-GFP. (C) Representative cochlear whole-mount immunofluorescence images obtained two weeks after PSCC injection. Hair cells were labeled with MYO7A (red), transduced cells were identified by native GFP fluorescence (green), and nuclei were labeled with DAPI (blue). GFP-positive MYO7A-positive IHCs were considered transduced, whereas OHCs showed no detectable GFP signal. Scale bars, 10 μm.

### 3.3 Expression of CasRx in the adult mouse cochlea

Having confirmed that AAV8-CMV-GFP efficiently induced GFP expression in hair cells, we next evaluated whether AAV8-CMV-CasRx-Flag-GFP (AAV8-CasRx) could mediate CasRx expression in vivo. AAV8-CasRx was injected through the PSCC into the inner ears of 4‐week‐old C57BL/6J mice. Two weeks later, whole‐mount immunostaining of cochleae revealed robust Flag expression in IHCs along the cochlear turns, whereas OHCs were Flag negative (Fig. 3B), demonstrating successful delivery and expression of CasRx in the inner ear. Nearly 100% of IHCs in the apical and middle turns expressed Flag, while in the basal turn approximately 10 of 25 IHCs per field were Flag positive, which may modestly reduce RNA editing efficiency in basal hair cells in future therapeutic applications. To assess the safety of viral injection, we compared hair-cell numbers in AAV8-CasRx-injected mice with those in age-matched untreated control mice. Cochlear whole-mount immunostaining showed no obvious difference in the numbers of IHCs or OHCs between the two groups (Fig. 3A and B).

**Fig. 3:**
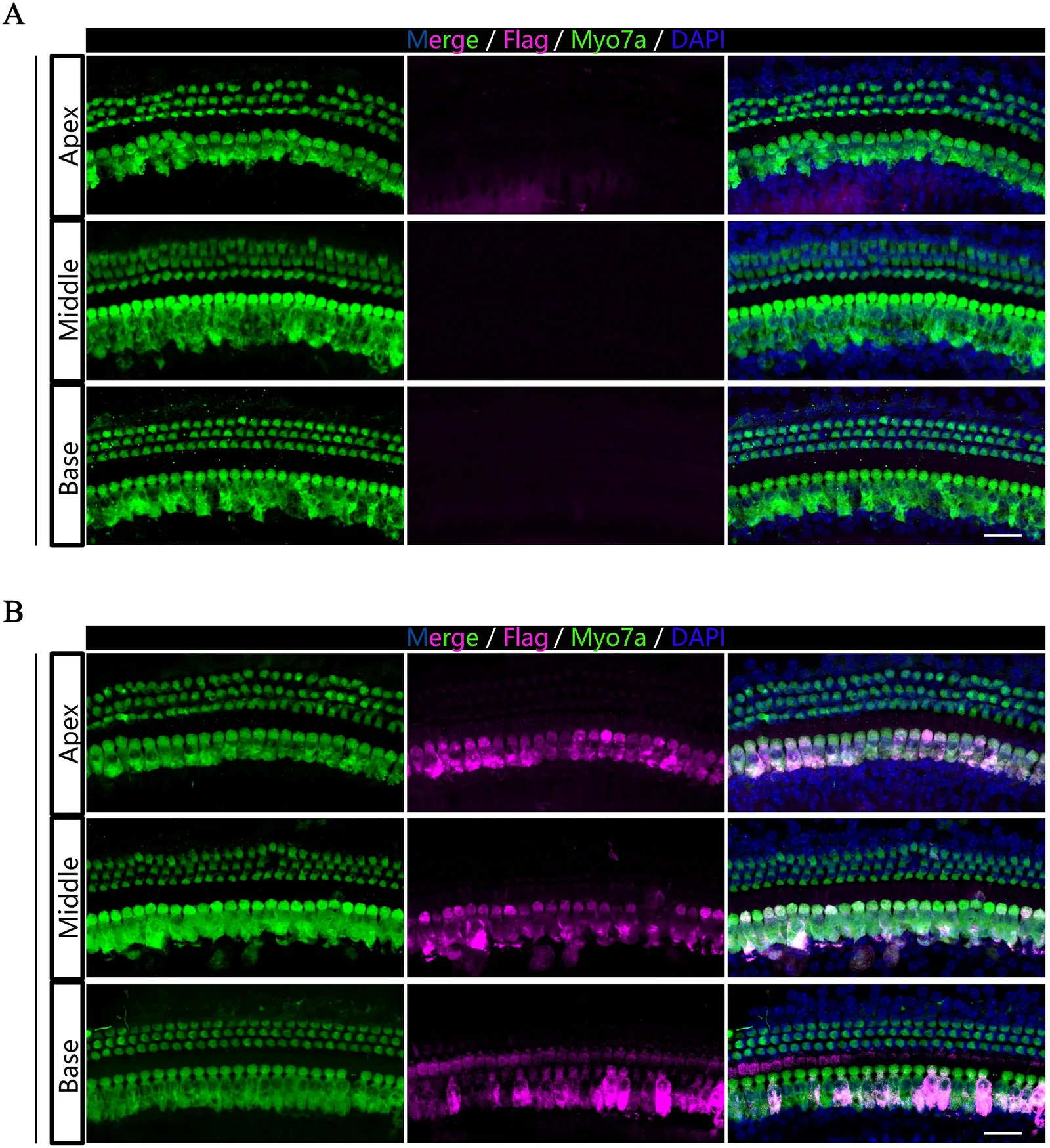
Hair-cell preservation after AAV8-CMV-CasRx-Flag-GFP injection into the mature cochlea. (A) Representative cochlear whole-mount immunofluorescence images from the apical, middle, and basal turns of untreated control mice. (B) Representative cochlear whole-mount immunofluorescence images from the apical, middle, and basal turns of AAV8-CMV-CasRx-Flag-GFP-injected mice. Hair cells were labeled with MYO7A (green), CasRx-expressing cells were detected by Flag immunostaining (magenta), and nuclei were labeled with DAPI (blue). No obvious loss of inner hair cells or outer hair cells was observed after AAV8-CasRx injection. Scale bar, 10 μm.

### 3.4 CasRx expression in the cochlear nucleus of adult mice

Although gene editing technology has been widely used along the auditory pathway, application to the cochlear nucleus has not been reported. The CN is crucial for auditory discrimination and perception and plays an important role in tinnitus generation [26]. We therefore investigated whether AAV8 could deliver and express CasRx in CN cells as an initial step toward future CN-targeted transcript-modulation studies. AAV8-CMV-CasRx-Flag-GFP was injected into the CN of 4‐week‐old C57BL/6J mice. Two weeks later, whole‐mount immunostaining of the CN demonstrated robust Flag expression in neurons and axons throughout the three subdivisions of the CN-DCN, posteroventral cochlear nucleus (PVCN), and anteroventral cochlear nucleus (AVCN)-at low magnification (Fig. 4B). At higher magnification, a subset of NeuN + neurons displayed Flag labeling, suggesting successful neuronal transduction. CasRx expression was also observed in many DAPI + NeuN-cells in the DCN, indicating that glial or other non‐neuronal cell types were transduced as well. These results demonstrate that AAV8-CMV-CasRx-Flag-GFP can be successfully delivered to the adult mouse CN and mediate detectable CasRx expression in both neuronal and non-neuronal cell populations, supporting the feasibility of this system as a delivery platform for future CN-targeted RNA-modulation studies.

**Fig. 4:**
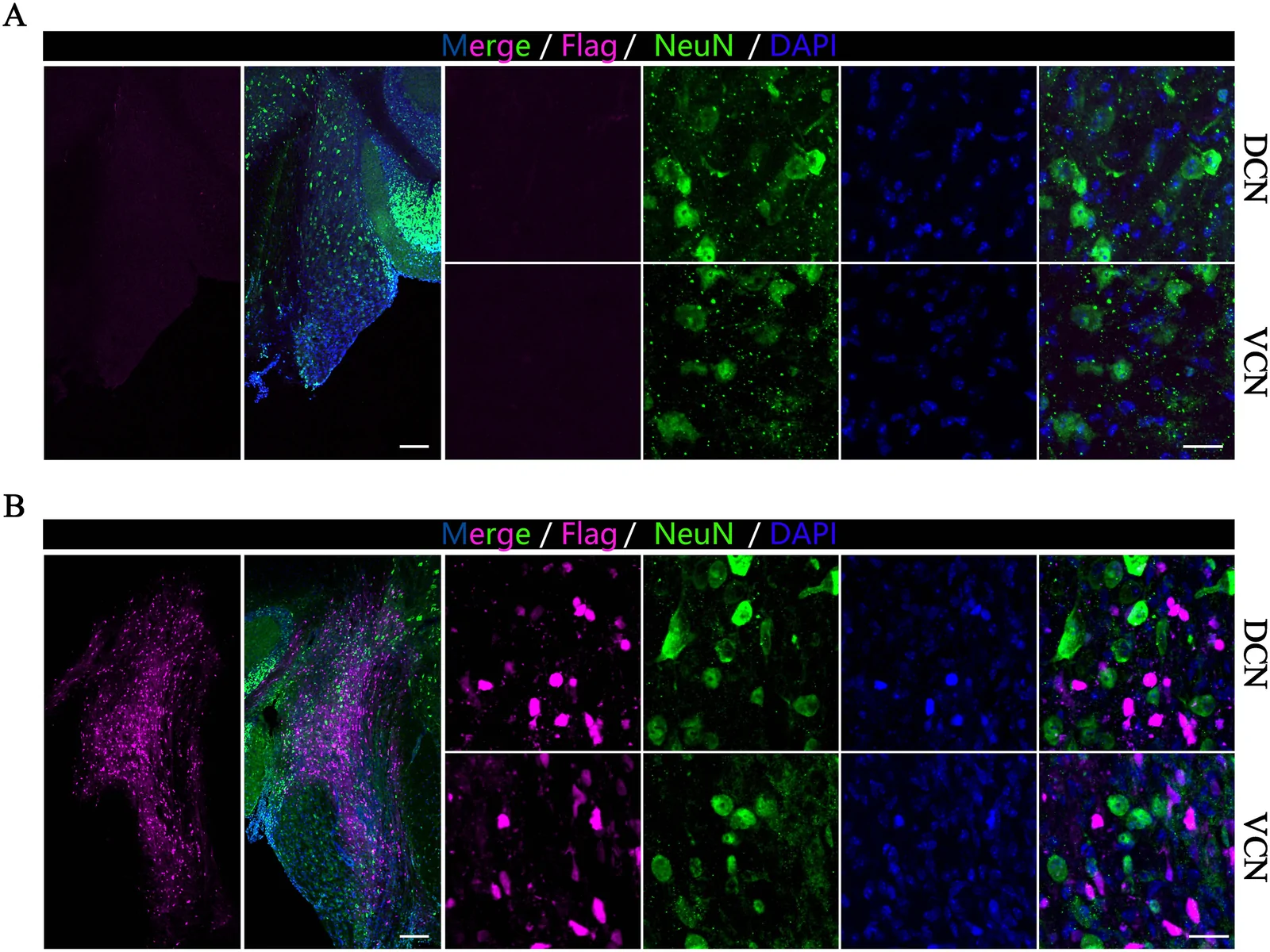
**AA8 mediated CasRx expression in CN of adult mice.** (A) Immunofluorescence of un-injected CN. (B) AAV8-CMV-CasRx-Flag-GFP transduced CN in adult C57 mice. Two weeks after injection, frozen slice immunofluorescence showed AA8-CMV-CasRx infection in the CN and mediated CasRx expression. Neurons were labeled with NeuN (Green) and infected cells were identified by Flag (Magenta). Scale bars, 100 μm for left; 20 μm for right. Representative CN sections from n = 3 mice per group are shown.

### 3.5 AAV8-mediated CasRx delivery and expression do not affect auditory thresholds in adult mice

To determine whether AAV8 infection of the cochlea and CN affects auditory function, we performed ABR tests two weeks after injection of AAV8–CMV–CasRx–Flag–GFP. No detectable threshold shifts were observed in AAV8‐injected ears compared with un‐injected control ears, indicating preservation of normal hearing (Fig. 5A, B, 5D). In cochlear‐injected mice, ABR thresholds were 38.3 ± 2.9, 28.3 ± 2.9, 15.0 ± 5.0, 20.0 ± 5.0, 25.0 ± 0, and 46.7 ± 5.8 dB SPL at 5.66, 8, 11.31, 16, 22.63, and 32 kHz, respectively (n = 3; Fig. 5B), which were comparable to the thresholds of un‐injected mice (37.5 ± 2.9, 25.0 ± 0, 16.3 ± 4.8, 17.5 ± 5.0, 21.3 ± 4.8, and 38.8 ± 4.8 dB SPL at 5.66, 8, 11.31, 16, 22.63, and 32 kHz; n = 4) and CN‐injected mice (37.0 ± 4.5, 26.0 ± 2.2, 16.0 ± 6.5, 14.0 ± 2.2, 22.0 ± 2.7, and 33.0 ± 2.7 dB SPL at 5.66, 8, 11.31, 16, 22.63, and 32 kHz; n = 5; Fig. 5D). The average wave I amplitude of ABRs in un‐injected mice tested at 80 dB SPL was 4.2 ± 1.3 μV at 16 kHz, similar to that in cochlear‐injected mice (4.3 ± 1.6 μV at 16 kHz; n = 3; Fig. 5C) and CN‐injected mice (3.8 ± 0.6 μV at 16 kHz; n = 5; Fig. 5E). These findings indicate that AAV8-mediated CasRx delivery and expression in the cochlea and CN did not adversely affect auditory thresholds in adult mice during the short-term observation period.

**Fig. 5:**
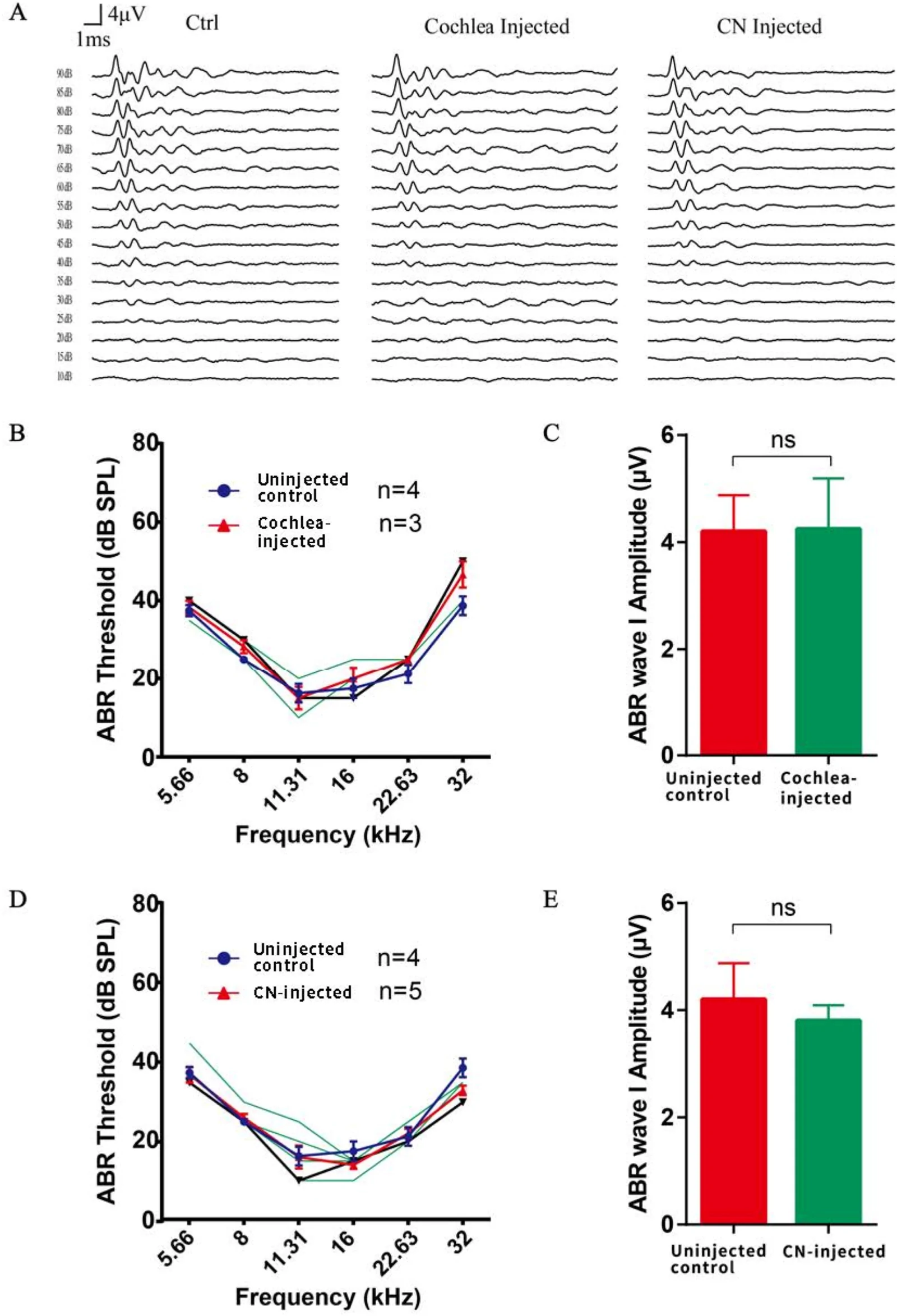
**Auditory function after AAV8-mediated CasRx gene transfer into the cochleae and CN of 4w C57 mice.** (A) Representative ABR waveforms at 16 kHz from uninjected control mice, cochlea-injected mice, and cochlear nucleus-injected mice. No obvious change in ABR waveform was observed after injection. (B) ABR thresholds for tone-burst stimuli at 5.66, 8, 11.31, 16, 22.63, and 32 kHz two weeks after cochlear injection. Thin green lines represent individual mice. Data are shown as mean ± SEM. (C) ABR wave I amplitude at 16 kHz two weeks after cochlear injection. (D) ABR thresholds for tone-burst stimuli at 5.66, 8, 11.31, 16, 22.63, and 32 kHz two weeks after cochlear nucleus injection. Thin lines represent individual mice. Data are shown as mean ± SEM. (E) ABR wave I amplitude at 16 kHz two weeks after cochlear nucleus injection.

## 4 Discussion

In this study, we constructed a CasRx expression plasmid containing a CMV promoter, CasRx coding sequence, Flag tag, and EGFP reporter and validated its expression in HEK293T cells by immunofluorescence and Western blot (Fig. 1C, A, 1D). We then packaged this expression cassette into an AAV8 vector and delivered it to the cochlea and CN of adult mice. AAV8 specifically targeted IHCs in the inner ear (Fig. 2C), and AAV8–CasRx efficiently mediated CasRx expression in both the cochlea and CN (Fig. 3, Fig. 4B). Two weeks after injection, ABR thresholds and wave I amplitudes remained unchanged, indicating that CasRx expression does not impair cochlear or CN function (Fig. 5A–F). Collectively, these findings indicate that AAV8-mediated CasRx delivery and expression in the adult cochlea and CN are feasible and do not detectably impair auditory function during the short observation period.

More than 100 genes have been implicated in hearing loss, many of which are critical for cochlear function [34], [35]. AAV‐mediated delivery of gene therapy constructs to the cochlea is therefore an attractive strategy to repair pathogenic variants and improve hearing [36]. Commonly used AAV serotypes, such as Anc80L65 and AAV9–PHP.B, can infect multiple cochlear cell types, including IHCs, OHCs, and supporting cells [19], [37]. However, some hereditary hearing losses are primarily caused by lesions restricted to IHCs, and a lack of vector specificity may increase the risk of off‐target effects in non‐diseased cells. For this reason, we chose AAV8 as the delivery vector in the present study. We observed that nearly all IHCs were infected in adult mice (Fig. 2C), and AAV8 showed excellent specificity and safety in IHCs, consistent with previous reports [33]. The observed distribution is consistent with previous reports of AAV8 transduction in the adult inner ear. This cellular pattern is most likely a consequence of the vector system and delivery route, including the AAV8 capsid, CMV promoter, PSCC injection, and local cochlear environment, rather than an intrinsic property of CasRx. Thus, our findings should be viewed as evidence for the feasibility of AAV8-mediated CasRx delivery and expression in the cochlea, but not as evidence that CasRx confers cochlear cell tropism. Earlier studies reported that AAV8 infected approximately 80% of IHCs in adult mice [34], which may reflect differences in viral purity, concentration, and stability across laboratories and production facilities [38]. While AAV8-mediated transduction of the adult inner ear has been described previously, its use for delivering CasRx to the mature cochlea has not been extensively evaluated. Here, PSCC delivery of AAV8-CasRx resulted in robust CasRx expression in IHCs, with no evident impairment of cochlear morphology or short-term auditory function. These observations indicate that AAV8 can serve as a feasible vector for CasRx delivery and expression in the adult cochlea. Nevertheless, in the absence of a target-specific guide RNA and direct transcript-level validation, these results should not be interpreted as evidence of functional RNA editing or transcript modulation.

CRISPR technology has greatly accelerated the development of gene therapy in the inner ear [39]. Gao et al. delivered a Cas9-gRNA ribonucleoprotein complex targeting the mutant Tmc1 Beethoven (Tmc1^Bth) allele in neonatal mice and protected auditory function during subsequent hearing development [18]. However, because CRISPR/Cas9‐based editing permanently modifies DNA sequences, off‐target events in normal genomic regions may irreversibly disrupt gene function [39]. In addition, the lack of cell‐type specificity for many vectors can result in widespread distribution of the gene editing machinery, leading to unintended genomic changes in adjacent cells and even distant tissues [24]. On this basis, we assessed the capacity of an AAV8-based vector to deliver CasRx to IHCs in the mature cochlea. PSCC administration resulted in readily detectable CasRx expression in IHCs, without apparent loss of IHCs or OHCs or obvious disruption of cochlear morphology. These results indicate that AAV8 is suitable for CasRx delivery and expression in the adult cochlea. Nevertheless, because no target-specific guide RNA or ADAR-based editing module was incorporated, these findings establish delivery and expression feasibility, rather than functional RNA editing.For future therapeutic applications, sustained CasRx and guide RNA expression may be required because target mRNAs are continuously transcribed. In the present study, we assessed CasRx expression and auditory safety only at two weeks after AAV delivery. Therefore, the persistence of CasRx expression in IHCs and CN cells, as well as the duration of guide RNA-mediated transcript modulation, remains to be determined. Future studies should include longer follow-up periods and direct measurements of target transcript regulation and specificity.

Previous studies have demonstrated that noise exposure can damage the cochlea, DCN, and VCN [40], [41], [42]. Animals with behavioral evidence of tinnitus often show increased SFR in DCN fusiform cells, which is considered a key neural correlate of tinnitus, whereas animals without tinnitus do not exhibit this pathological hyperactivity [26]. Thus, tinnitus onset and persistence are closely associated with DCN pathophysiology. Several current strategies aim to treat tinnitus by targeting DCN activity. For example, pharmacological activation of KCNQ channels can prevent tinnitus development and promote reduction of aberrant HCN channel activity, but no EMA‐ or FDA‐approved drugs are currently available [43], [44].

RNA-targeting strategies may offer a future route for manipulating disease-relevant transcripts in auditory circuits, including the CN[47-49]. Here, we focused on the preceding delivery step and assessed whether AAV8 could mediate CasRx expression in the adult mouse CN. Flag labeling was detected throughout the DCN and much of the VCN two weeks after injection and was present in both NeuN-positive neurons and DAPI-positive/NeuN-negative cells, indicating transduction of multiple CN cell populations (Fig. 4). These findings establish the feasibility of AAV8-mediated CasRx delivery and expression in the CN. However, because no target-specific guide RNA or transcript-level assay was included, further studies will be required to determine whether this approach can achieve selective RNA modulation and functional effects in CN circuits[50-52].

This study has some limitations. First, we only evaluated the safety of CasRx expression in the cochlea and CN and did not perform therapeutic interventions in disease models. Future work will apply this system to specific deafness and tinnitus models to assess functional rescue. Second, due to the lack of specific antibodies against CasRx, we inferred CasRx expression using the Flag tag rather than direct detection of CasRx protein. Nevertheless, Flag tagging is a standard and widely accepted approach for tracking protein expression, and our conclusions remain well supported. Another limitation of this study is that ABR acquisition and threshold calling were not performed under blinded conditions, although the same predefined criteria were applied consistently across all groups. In addition, only male mice were used in this study; therefore, possible sex-dependent differences in AAV8 transduction efficiency, CasRx expression, and auditory safety were not evaluated. Future studies should include both male and female animals. Taken together, our findings establish a preliminary delivery and expression framework for AAV8-mediated CasRx application in the cochlea and cochlear nucleus. Although functional RNA modulation was not examined in the present study, these data provide a useful foundation for subsequent work incorporating target-specific guide RNAs, ADAR-based editing modules, and rigorous transcript-level and functional validation in auditory disease models.

In summary, our data demonstrate that AAV8 can efficiently mediate CasRx expression in IHCs of adult mice and that AAV8-CasRx can be delivered to both the inner ear and CN without compromising auditory function in the short term. These findings demonstrate the feasibility and short-term safety of AAV8-mediated CasRx delivery to auditory structures and provide a platform for future studies using guide RNA-directed transcript modulation in deafness, tinnitus, and other auditory disorders.

## Author contributions

Shuang Zhou: Conceptualization, Methodology, Investigation, Data curation, Formal analysis, Visualization, Writing-original draft. Tao Huang: Methodology, Investigation, Formal analysis, Validation. Wei Yao: Investigation, Methodology, Visualization. Shan Huang: Investigation, Validation, Data curation. Junjie Jiang: Investigation, Validation. Hua Liao: Conceptualization, Resources, Funding acquisition, Supervision, Project administration, Writing,-review and editing. Zhongfang Xia: Conceptualization, Resources, Supervision, Project administration, Writing-review and editing. All authors reviewed and approved the final manuscript.

## Ethics declaration

This study was conducted in accordance with the ARRIVE (Animal Research: Reporting of In Vivo Experiments) guidelines. This study was approved by the Animal Care and Use Committee of the People's Hospital of Wuhan University. (Approval No. 20220301A)

## Declaration of generative AI and AI-assisted technologies in the writing process

During the preparation and revision of this work, the authors used ChatGPT (OpenAI) to assist with language polishing and organization of the manuscript. After using this tool, the authors reviewed and edited the content as needed and take full responsibility for the content of the published article.

## Declaration of competing interest

The authors declare that they have no known competing financial interests or personal relationships that could have appeared to influence the work reported in this paper.

## Data Availability

All data generated or analyzed during this study are included in this article. Raw ABR traces, original immunofluorescence images, and Western blot source images are available from the corresponding author upon reasonable request. No publicly available datasets were generated or analyzed in the present study. (Mendeley Data, V1, doi: 10.17632/bptryr3h3f.1).
